# The biology of cultural conflict

**DOI:** 10.1098/rstb.2011.0307

**Published:** 2012-03-05

**Authors:** Gregory S. Berns, Scott Atran

**Affiliations:** 1 Center for Neuropolicy, Emory University, Atlanta, GA 30322, USA; 2 CNRS-Ecole Normale Supérieure, Institut Jean Nicod, 29, Rue d'Ulm, 75005 Paris, France

**Keywords:** culture, neuroscience, functional magnetic resonance imaging, religion, politics

## Abstract

Although culture is usually thought of as the collection of knowledge and traditions that are transmitted outside of biology, evidence continues to accumulate showing how biology and culture are inseparably intertwined. Cultural conflict will occur only when the beliefs and traditions of one cultural group represent a challenge to individuals of another. Such a challenge will elicit brain processes involved in cognitive decision-making, emotional activation and physiological arousal associated with the outbreak, conduct and resolution of conflict. Key targets to understand bio-cultural differences include primitive drives—how the brain responds to likes and dislikes, how it discounts the future, and how this relates to reproductive behaviour—but also higher level functions, such as how the mind represents and values the surrounding physical and social environment. Future cultural wars, while they may bear familiar labels of religion and politics, will ultimately be fought over control of our biology and our environment.

## 1. Cultural conflict and why biology matters

In the most general sense, culture can be thought of as the knowledge, customs and traditions of a group of people [1], which systematically drive and channel collective dispositions of thoughts and behaviours into the future. Culture includes social, legal and economic institutions, as well as non-institutionalized trends and movements. Culture encompasses technology, literature and art, as well as disparate political, ethnic and religious beliefs and biases that both infuse and connect the higher cognitive functions and emotions of individual brains [2].

Although culture is usually thought of as the collection of knowledge and traditions that are transmitted outside of biology, one cannot credibly deny that the thoughts and behaviours of individuals contribute to the creation of culture, and that every person must process and react to cultural phenomena. Over 100 years ago, William James said it clearly, ‘There is not a single one of our states of mind, high or low, healthy or morbid, that has not some organic process or condition… They [beliefs] are equally organically founded, be they of religious or non-religious content’ [3, p. 16].

Thus, cultural conflict should manifest in two ways. First, if there are systemic and substantial cultural differences between groups of people, this would result in different types of processing in individual brains that form the group. Take, for example, religion. When presented with a concept like God, a Christian and an atheist would surely react differently, and this will probably manifest as differences in brain activation [4]. Similarly, in the US political realm, probing the role of government spending could well elicit different brain activations for Republicans, Democrats and Tea Party members. Second, mere cultural differences in brain activation do not necessarily imply conflict. Cultural conflict would be hypothesized to occur only when certain beliefs and traditions of one culture represent a challenge to individuals of another culture. Such a challenge would elicit brain processes involved in the cognitive decision-making, emotional activation and physiological arousal associated with the outbreak, conduct and resolution of conflict.

Because biological processes govern our perceptions, interpretations and reactions to cultural events, understanding these processes will not only help us understand cultural conflicts but also potentially mitigate them. In this issue, we have collected a series of papers that begins to tackle issues surrounding cultural conflict from a biological perspective. The cultural themes range from political partizanship to sacred values and religious conflicts, and the tools used to study them include brain imaging with functional magnetic resonance imaging and measures of physiological arousal (skin conductance responses (SCRs) and eye-tracking).

## 2. Primitive drives

We begin with the most primitive biologic processes linked to decision-making: good versus bad. Every animal makes decisions about things that it wants and things it avoids. In human economics, we designate these categories as ‘goods’ and ‘bads’, but behaviourally these categories can be mapped out by things that individuals approach or avoid. For humans, there are certain universals. We generally like (and approach) things linked to survival and prosperity: food, mates and money; and we generally dislike (and avoid) things linked to mortality and loss. Although universal, cultural differences shape their relative importance to individuals, and so we begin by examining responses to these biologically primitive drives. For example, which is more important—seeking out the good things or avoiding the bad? Differences over this basic decision may cause conflicts both within and between cultures. Dodd *et al*. [5] approach the question in terms of political affiliation.

Even within a society, individuals may hold different beliefs about politics that lead to cultural conflict. Strictly defined, politics refers to governing institutions and policies. However, political affiliations often align with other cultural and religious beliefs, so that when we talk about political differences, these may include broad cultural differences even within a society. There appears to be a strong disposition to categorize in terms of binary oppositions: to dichotomize [6], essentialize [7] and thereby deepen outward differences that may have initially been superficial or arbitrary. Ever since the French Revolution, it is common to divide secular political camps into the ‘left’ and ‘right’. The left/right division has different meanings in different countries but generally maps onto bigger or smaller roles of government. In the USA, it is liberals and conservatives, or Democrats and Republicans. In the UK, Labour and Conservative parties; in France, left (e.g. Socialist Party) and right (e.g. RPR); in Germany, the left (SPD) and the right (CDU/CSU); in Spain, the left (PSOE) and the right (PP); in Israel, Labor and Likud, and so on.

Do such divisions of left and right on the political spectrum merely reflect the human tendency to categorize, or might there be fundamentally two contrasting types of politically relevant cognitive and social dispositions that differentially characterize individuals in every culture? Dodd *et al*. [5] provide physiological evidence for the latter. Using SCRs, which are a measure of physiological arousal, they find significant differences between people on the left and the right. Importantly, the differences appear only when subdivided into ‘good’ and ‘bad’ provocateurs. Those on the right show arousal responses to pictures of aversive stimuli like maggot-ridden meat and angry mobs, while those on the left show arousal responses to positive pictures like rabbits and happy children. A follow-up study using eye-tracking to measure attention confirmed that attention and arousal are yoked together along these same dimensions.

These findings may help to explain differential support for policy differences between the political left and right. Individuals on the political right appear to be more sensitive and attuned to the unpleasant things in life. As Dodd *et al*. [5] note, ‘this responsiveness, in turn, is consistent with the fact that right-of-centre policy positions are often designed to protect society from out-group threats (e.g. by supporting increased defence spending and opposing immigration) and in-group norm violators (e.g. by supporting traditional values and stern penalties for criminal behaviour)’. If true, then the rules and policies advocated by the two poles of the political spectrum are there to mitigate biological sensitivity to unpleasantries.

Another primitive biological process that all animals must face is how to value the future. Humans have extensive cognitive capacity for both remembering the past and imagining the future, and how we value the future has ramifications for individuals and societies. When the future is expected to be better than the present, there is motivation to invest in the future. Such investments include having children, emphasizing their education, investing and building infrastructure, saving for retirement and adopting behaviours that prolong and increase the quality of life. On the other hand, when the future is expected to be worse than the present, the incentives move towards living in the present: profligate consumption and reduced infrastructure investment.

One way to measure the value of the future is through an individual's discount rate. This is the rate at which time devalues future expected values for that individual. Kim *et al*. [8] examine biological differences in discount rates between Koreans and Americans. They find that Americans have discount rates over twice that of Koreans, and that these differences are mirrored in the activity of the ventral striatum—a brain structure well-known to be associated with value-based decisions. These findings lay the groundwork for understanding differences in culturally situated beliefs towards savings and investment, which may be a source of conflict.

Another biological primitive, which may also relate to future discounting, is reproductive behaviour. Henrich *et al*. [9] examine the cultural conditions that foster and inhibit monogamous marriage. Like discount rates, a society's institutions for marriage provide a window into how the culture values the future. Fundamentally, marriage is a framework that allows society to recognize reproductive rights, and secondarily, to provide for an orderly passing of property to offspring. Although marriage is a cultural institution, reproduction is generally expected to be a consequence of the arrangement, and therefore, intertwined with biology. Given that males can reproduce with relatively low cost, and that historically 85 per cent of societies have allowed men to have multiple wives, how could monogamy ever be adaptive? Henrich *et al*. [9] suggest a theory with a simple premise: polygamy creates a residual pool of males with no possibility of having a wife. With limited prospects of future reproductive success, these males should have steeper discount rates (substantially higher valuation of the present), which is associated with more impulsive behaviours: criminal activity, violence and drug use. Henrich *et al*. [9] argue that these are destabilizing influences in a society. Adopting monogamy as the cultural norm ensures a mate for everyone, and crime and violence decrease, benefitting all. In contrast, polygamous societies will have a large pool of males with no hope for reproduction. These males can be channelled into armies and sacrifice their genes for ‘their brothers’.

Carrying the theme of conflict forward to violent means, there is considerable historical, cross-cultural and psychological evidence that males and females differ in aggressive tendencies, especially in the most violent behaviours of aggravated assault and homicide [10], war and terrorism [11]. McDonald *et al*. [12] propose an evolutionary-based argument for why this is the case. It has been suggested that females are a resource for which males aggressively compete. However, ‘this competition need not take the form of direct contests for instances of sexual access, but may include conflicts over feeding territories, nest sites and more intangible resources, such as social influence, power and status—resources that can be converted into reproductive opportunities over time’. They suggest that intergroup conflict has affected the social psychologies of men and women differently. Because men are the more common perpetrators and victims of intergroup aggression, coalitional psychology is likely to be more pronounced among men. From this, McDonald *et al*. [12] argue that selection has favoured the evolution of cognitive processes for ‘the formation of male coalitions capable of planning, initiating and executing attacks on out-groups with the aim of acquiring or protecting reproductive resources’, which is referred to as the ‘male warrior hypothesis’.

## 3. ‘Give me liberty or give me death’

In *The Origin of Species*, Charles Darwin considered adaptations—including warlike and altruistic behaviour in humans—only for the individual's own use in its struggle to gain resources to produce offspring: ‘good for itself’, but ‘never … for the exclusive good of others’ [13, p. 230]. Later, however, he puzzled over the problem of how self-interest alone could account for humankind's aptitude for self-sacrifice to the point of giving up one's life—the totality of a person's self interests—for tribe, nation, religion or for humanity. The puzzle led Darwin to modify his view that natural selection only produces selfish individuals. In *The Descent of Man*, he suggested that humans have a naturally selected propensity to the virtue of ‘morality’, that is, a willingness to sacrifice self-interest in the cause of group interests. This includes heroism in battle, and martyrdom, where prospects for personal survival are very low but somewhat higher for those in the group who may be neither kin nor kith. Groups possessing an abundance of individuals with such moral virtue, Darwin argued, would be better endowed in history's spiralling competition for survival and dominance [14].

The nature of moral values is, in large part, defined by the culture in which individuals engage them in decisions, but virtue theory suggests two very different ways in which moral values might be processed [15]. Moral values could be either deontological in nature [16] or they could be utilitarian [17]. Deontic processing is defined by an emphasis on absolute rights and wrongs, whereas utilitarian processing is characterized by costs and benefits. Models of rational behaviour predict many of society's patterns, such as favoured strategies for maximizing profit or likelihood for criminal behaviour in terms of opportunity costs [18]. But the prospects of crippling economic burdens and huge numbers of deaths do not necessarily sway people from their positions on whether going to war, or opting for revolution or resistance, is the right or wrong choice [19]. One possible explanation is that people are not weighing the pros and cons for advancing material interests at all, but rather using a moral logic of ‘sacred values’—convictions that trump all other considerations—that cannot be quantified in straightforward ways [20].

In potentially violent situations of intergroup conflict, sacred values appear to operate as moral imperatives that generate actions independently, or out of proportion, to their evident or likely results, because it is the right thing to do whatever the consequences [21]. For example, regardless of the utilitarian calculations of terror-sponsoring organizations, suicide terrorists appear willing to make extreme sacrifices that use a ‘logic of appropriateness’ rather than a calculus of probable costs and benefits [22]. Or consider the American revolutionaries who, despite belonging to a society that had the highest standard of living in the world, defied the greatest empire, army and navy of the age in pledging ‘our lives, our fortunes, our sacred honour’ for the cause of ‘liberty or death’, where the desired outcome was highly improbable by any measure of manpower or available means of material warfare [23].

The problem with sacred values, from an experimental point of view, is that they are difficult to study in the laboratory. Berns *et al*. [24] describe a novel paradigm in which they use integrity as a proxy for the strength of an individual's commitment to a particular cultural value. Integrity refers to an individual's consistency of values and actions. For example, although we cannot test whether an individual is willing to kill an innocent human being (a common cultural taboo), we can test their willingness to sign a document that says they would. Although signing such a document does not bind the person to that action, it creates an inconsistency between value and action that signals a loss of integrity. It is reasonable to assume that if something is truly sacred, then an individual would maintain their integrity for that value and not sign such a document. What if they were offered money to sign? It then becomes a trade-off between the monetary gain and the cost in personal integrity.

If sacred values are represented in a utilitarian manner, then prior neuroeconomic research suggests that they should be associated with increased neural activity in brain regions associated with the calculation of utility; alternatively, if sacred values are represented as deontic rules, then brain regions associated with the processing of moral permissibility (rights and wrongs). Interestingly, Berns *et al*. [24] find evidence for the deontic processing of sacred values. Moreover, they find that the stronger the deontic processing in brain regions associated with the engagement of rules, the more active an individual tends to be in group organizations. This suggests that groups carry and inculcate cultural rules in the brains of individuals.

Cultural conflict is likely to emerge when the rules and values of one cultural group are substantially different from another, and members of the cultures come in contact with each other. How individuals react depends greatly on the specific context, but the findings in this issue point to generic biological mechanisms. As Berns *et al*. [24] show, the amygdala—a key structure for physiological arousal—is activated when individuals are presented with statements contrary to their own personal sacred values. Although amygdala activation is not specific for a particular emotional state, it is consistent with heightened arousal. But in a conflict situation, it is most likely a negative emotional state of high arousal. This is important because this is the physiological state associated with ‘fight or flight’. Confronting individuals' sacred cultural values with conflicting ones, places the individuals in a state in which they are more likely to experience ‘moral outrage’ and engage in violence [25].

One constellation of values that appears to acquire sacred status in a variety of different cultural settings, and whose violation often generates moral outrage that can lead to extreme violence, concerns the conception of ‘honour’ [26]. Gelfand *et al*. [27] discuss the importance of honour in Middle Eastern countries. They find that in Middle Eastern cultures honour is not only a status indicator for individuals, but that it is a transferable resource to immediate family members. Moreover, honour is a shared resource with ‘ripple effects on the extended family, friends and social circles, the community, neighbourhood, tribe and organizations’. When honour is lost through the actions of an individual, the extended community suffers. Thus, there is a strong incentive for the establishment of cultural rules that treat honour as a sacred value. Any perceived violation of the code of honour by those outside the society may be grounds for violence and even war [28], whereas violation by individuals within a culture of honour may be considered an attack upon the moral foundation of the society that merits extreme punishment [29].

## 4. Enforcement of cultural rules

Social groups that affirm and maintain their identity through cultural rules must also have the means to enforce compliance. Like the primitive drives noted earlier, enforcement mechanisms must be either rewarding or punishing in nature. Rewards for group membership can be explicit through recognition and conferring of status vis-à-vis titles; through conspicuous displays of status in the form of material wealth or number of children, for example; or indirectly through reciprocal relationships with other members of the group—for example, business deals or marriages. Punishments, on the other hand, diminish social status by taking away the opportunity to reap these rewards. Punishments can be explicit and public, e.g. prison or corporal punishment, or implicit through shunning and loss of relationships within the community, which closes the opportunity to do business or have a spouse.

Huettel & Kranton [30] address this relationship between individuals and their social groups by suggesting a new framework based on ‘identity neuroeconomics’. They adapt the standard expected utility model of decision-making to include a cultural term that interacts with individual utility. In this model, ‘identity utility’ depends on the extent to which one's own and others' actions match prescribed behaviour. Identity utility also depends on the status of one's social group, and the match between the individual's attributes to the ideal of the social group. Whether it is honour or status or material markers of status, their framework suggests ways in which one might measure how culture affects individual decision-making.

Along these lines, the way culture affects the individual can be measured in the laboratory by controlling specific elements of culture. Kishida *et al*. [31] do exactly this by creating an experimental culture in which status is defined by performance on an intelligence test. In many cultures, intellectual achievement is a marker of status and success, and so this is a reasonable place to start. Specifically, they explore the neural effects of publicly broadcasting this status marker. Behaviourally, they find that broadcasting ranks of intelligence globally depressed everyone's performance, and only a subset of individuals were able to recover. The implication is that broadcasting social rank, whether by intelligence or some other metric, is a powerful tool to both reward and punish culturally sanctioned behaviours. Kishida *et al*. [31] shows that the biological effect of cultural enforcement may lie in the amygdala. Individuals who are able to inhibit the amygdala, through activation of the left prefrontal cortex, may be relatively immune to cultural norms. If so, this may ultimately shed light on what types of individuals comply with cultural norms, resist them or react violently when the norms are threatened.

## 5. From differences to conflict

Just because cultures are different does not necessarily mean they will end up in conflict. Thus, while cultural differences may be a facilitating condition for conflict to occur, differences alone are insufficient. The same logic applies to biological differences: the mere demonstration of biological differences between cultural groups does not mean that a conflict will follow. As noted above, cultures manifest a variety of mechanisms to instill and maintain their internal set of beliefs, which, when challenged, set in motion a series of physiological responses that prime individuals for violent action. Who engages in violence and who approaches conflict from the standpoint of negotiation?

Two papers in this issue examine brain responses across cultural groups already in conflict and provide important new insights into the cognitive processes evoked when individuals are forced to consider the perspectives and beliefs of someone that, in other circumstances, might be considered an enemy. The advantage of studying members of groups already in conflict is that they provide a cross-sectional snapshot of both cognitive and emotional responses to established in- and out-groups.

Bruneau *et al*. [32] suggest that when groups are in conflict, cultural biases serve to further drive the groups apart and prevent reconciliation. They theorize that these biases inhibit the individual's capacity to either mentalize about the states of mind of someone from the conflicting culture or empathize with their pain. Using Arab and Israeli subjects, they examine the neural circuits associated with processing poignant stories of members of the corresponding in- and out-groups. If these longstanding cultural conflicts have resulted in an inability to empathize the pain of the opposing group, then, as Bruneau *et al*. [32] suggest, this should lead to blunted responses in the brain's pain matrix to depictions of pain in the opposing group. Although a variety of behavioural metrics are consistent with warmer feelings towards the in-group, and less empathy for the out-group, the neuroimaging results suggest a more nuanced explanation.

Responses in brain regions associated with mentalizing were equally large for both Arab and Israeli participants reading about Israeli and Arab targets, but less so for a distant, third-party group (South Americans). This suggests that the brain processes associated with mentalizing have more to do with the salience and proximity of the group rather than ‘friend’ or ‘enemy’ labels. More than these labels, empathic responses may be driven by personal significance. This dovetails with Gelfand's results, suggesting that personal salience can be amplified by the construct of honour, especially as it can be shared.

Another testbed of cultural conflict can be found in the USA between Democrats and Republicans, especially those who have strong party affiliations. As Dodd *et al*. [5] showed, skin conductance measures suggest differences in arousal to good and bad stimuli, thus setting the stage for a biologically mediated conflict between Democrats and Republicans. Examining the issue directly, Falk *et al*. [33] focus on brain responses in Democrats and Republicans in the months leading up to the 2008 presidential election. As they note, the election provides a focal point that increases the personal salience of whatever conflict is perceived between members of the two parties. Thus, whatever differences exist between Democrats and Republicans, an election forces them into conflict because only one can win. Falk *et al*. [33] had Democrats and Republicans consider issues from the stance of their own party's candidate or the other (McCain and Obama). Interestingly, they find that regions associated with mentalizing functions, especially the medial prefrontal cortex, were more active when taking the perspective of one's own candidate. Moreover, the effect was exaggerated in individuals who measured higher on scales of perspective taking. One of the presumed impasses to negotiation between conflicted groups is the inability to see things from the other side. As Falk *et al*. [33] note, even individuals who exhibit temperaments that are more empathic may deploy this ability selectively—an effect that was amplified as the election grew closer.

If the ability to empathize with, or take the perspective of, someone from an out-group is reflected in the responsiveness of prefrontal circuits, then what about trusting them? Stanley *et al*. [34] examine neural responses in a ‘trust game’ and how these responses are affected by the race of the individual to be trusted. In the trust game, participants are given an endowment of money, from which they can share with a trustee. Any money sent to the trustee is quadrupled, and then the trustee can either keep it or split the proceeds 50/50. The exchanges are anonymous, except that the participant is shown a picture of the partner's face before deciding how much to send. Racial bias can be measured by the difference in amounts of money sent to black versus white trustees. Stanley finds that the ventral striatum activity correlates with the individual's race bias: this structure was more active when making decisions about individuals from whichever race they trusted less. Although striatal activity is typically related to the expected value of outcomes, growing evidence suggests that the striatum also signals the salience of the action itself [35]. This is consistent with Bruneau's findings that groups in conflict with each other are highly salient to each other.

## 6. What does it mean?

The 50 years following World War II were a period of modern history that was unprecedented for its constancy in terms of the bipolar rivalry between global secular ideologies, and the dominance of a ‘rational actor’ paradigm for dealing with that rivalry. It seems increasingly obvious that such an era is over. As we noted earlier, cultural differences do not always lead to conflict, but several factors on both a local and global scale have increased the likelihood of conflict. A vastly increased population means more people competing for limited resources, and the globalization of the economy means that local conflicts ripple throughout the world, affecting markets and distribution of raw materials. Modern communication through text messaging, social networking and new Internet technologies ensure that news of conflict spreads almost instantly. Thus, where geographical remoteness previously had a strong role in keeping conflicts local, we are now in the situation where riots in Greece or Mumbai, for example, have immediate global consequences. Consequently, the two basic requirements for the initiation of cultural conflict—substantial differences in beliefs and active challenges to those beliefs—are now done electronically. Physical proximity is no longer a necessary condition for the engagement of the biological requirements for conflict.

Cultural conflicts are not simply the result of different traditions. The proverbial ‘clash of civilizations’ may be less appropriate as a characterization of post-Cold War conflicts throughout the world than a crisis, or even collapse, of traditional territorial cultures. Vertical, generation-to-generation forms of social structure and information hierarchies are breaking down and many, especially the young, are forming their identities in global, media-driven political cultures through horizontal peer-to-peer relationships that ignore historical and spatial constraints [36]. But whereas Internet communication and revivalist religious ideologies may increasingly serve as facilitators and vehicles for conflict, root causes may remain primitive and biologic. Fundamentally, people want to survive, prosper and create a better future for their children and those they care for, including genetic strangers that form part of primary reference groups, be it their tribe, nation, religion or conception of ‘humanity’. When these basic goals are threatened, conflict is more likely.

Many of the papers in this special issue deal with the way in which cultural differences map onto biological differences in the brain. We will set aside the question of causality and take these observations at face value. For example, biological differences in discount rates have direct implications for behaviour. All things being equal, a society in which individuals tend to have steeper discount rates will behave more impulsively. Because the future is worth relatively little, such cultures would resist investing in infrastructure; would tend to devalue education; would engage in more rapid depletion of their resources; and would generally ‘live for the moment’.

Just because there are biological differences does not mean they are immutable. We know, for example, that individual discount rates can be altered by drugs. Unfortunately, most of the documented effects of drugs, such as tobacco, are associated with increased discount rates, making individuals even more impulsive [37]. However, given evidence for the close link between discount rates and foraging behaviour in animals, it is possible that even simple changes in human nutrition would affect an individual's behaviour on a societal scale. Beyond calorie counts, how might different amino acids and fatty acids affect discount rates? Viewed through the lens of biology, dietary choices may be directly related to resource consumption, birthrates and violence simply by the effect of nutrition on the dopamine system and its discount rate for the future.

Another area for future inquiry is the possible effect of sacred values on discount rates. For example, people may perceive temporally distant but culturally significant events to actually feel closer in time than do more recent events, especially in contexts of group conflict: for example, important episodes in religious or national history. This may be especially salient when people visit, or think about, ‘sacred places’ that evoke significant cultural events, such as a hallowed shrine or battlefield. Evocation of these sentiments might have profound biological effects in the form of memory reactivation (good and bad) and physiological arousal, leading to fight or flight responses. Understanding these biological mechanisms helps us understand why one cultural group might be willing to invest in social infrastructure, while another wants to destroy it. Ultimately, biological responses determine who is ready to engage in war, and who wishes to seek peace.

As we begin to unravel the links between culture and biology, we are seeing how culture affects the brain. But what about the other direction? If the biology of the brain is changed, whether through diet, climate, chemicals or, inevitably, genetic engineering, will culture change? If, as we believe, culture and biology are yoked together, then future cultural conflicts will also play out biologically. Some cultures will embrace ways to change their biology and, in the process, change their culture. Others will reject such engineering. As a preview of what to expect, we might look to the conflicts that took place (and are still occurring) over contraception. Almost 100 years ago, Marget Sanger forcefully argued, ‘contraception needs no external justification—*it is a civilizing force in itself*, and carries with it its own immediate benefits, its own rewards to the parents, to the children, and to the community at large’ [38, p. 536]. The development of the birth control pill in the 1950s, set the stage for a full-blown cultural war over the right of women to control reproductive biology. Downstream cultural effects resulted in more women delaying marriage, going to college and entering the workforce [39]. Future cultural wars, while they may bear familiar labels of religion and politics, will ultimately be fought over control of our biology and our environment. The sooner we understand these relationships, the better position humankind will be in to mitigate these looming conflicts.
